# Zigzag persistence for coral reef resilience using a stochastic spatial model

**DOI:** 10.1098/rsif.2023.0280

**Published:** 2023-08-23

**Authors:** R. A. McDonald, R. Neuhausler, M. Robinson, L. G. Larsen, H. A. Harrington, M. Bruna

**Affiliations:** ^1^ Mathematical Institute, University of Oxford, Oxford OX2 6GG, UK; ^2^ Department of Geography, University of California, Berkeley, CA 94720, USA; ^3^ Computer Science Department, University of Oxford, Oxford OX1 3QG, UK; ^4^ Wellcome Centre for Human Genetics, University of Oxford, Oxford OX3 7BN, UK; ^5^ Department of Applied Mathematics and Theoretical Physics, Centre for Mathematical Sciences, University of Cambridge, Cambridge CB3 0WA, UK

**Keywords:** zigzag persistence, individual-based modelling, coral reefs, stochastic bifurcation, topological data analysis

## Abstract

A complex interplay between species governs the evolution of spatial patterns in ecology. An open problem in the biological sciences is characterizing spatio-temporal data and understanding how changes at the local scale affect global dynamics/behaviour. Here, we extend a well-studied temporal mathematical model of coral reef dynamics to include stochastic and spatial interactions and generate data to study different ecological scenarios. We present descriptors to characterize patterns in heterogeneous spatio-temporal data surpassing spatially averaged measures. We apply these descriptors to simulated coral data and demonstrate the utility of two topological data analysis techniques—persistent homology and zigzag persistence—for characterizing mechanisms of reef resilience. We show that the introduction of local competition between species leads to the appearance of coral clusters in the reef. We use our analyses to distinguish temporal dynamics stemming from different initial configurations of coral, showing that the neighbourhood composition of coral sites determines their long-term survival. Using zigzag persistence, we determine which spatial configurations protect coral from extinction in different environments. Finally, we apply this toolkit of multi-scale methods to empirical coral reef data, which distinguish spatio-temporal reef dynamics in different locations, and demonstrate the applicability to a range of datasets.

## 1. Introduction

Spatial patterns arise in many natural systems, from systems of chemical species or morphogens [1], cells in developing embryos [2,3], skin patterns on fish and mammals [4,5], and coral colonies in coral reefs [6]. Alan Turing explained the mechanisms behind the spatial patterns observed in morphogenesis—the interplay of diffusion and reactions [7]. Recent work has shed light on the importance of early segregation, or spatial patterning in embryos for successful development [8]. A common denominator of such systems is their complexity: they are dynamic, involve large numbers of particles or agents (e.g. molecules, cells, animals), and are inherently noisy. To elucidate the role of spatial patterns in such systems’ function and spatial evolution requires quantitative tools that can cope with such complexity. In recent years, the area of topological data analysis (TDA) has blossomed to offer multiple promising methods [9]. TDA can provide multiscale summaries of complex data. Here, we dive into the mechanisms of spatial patterning with TDA and other topological descriptors, with shallow-water coral reefs as a case study.

Coral reefs provide a tremendous range of ecosystem services, including biodiversity, fishing, and tourism [10,11]. Due to the complexity and stability of their calcium carbonate structure, specifically in shallow waters, coral reefs supply the optimal foundation for various photosynthetic benthic organisms to settle and grow upon. As such, there is relentless competition for space on the reef. Under human or natural disturbances—which are becoming ever more common with climate change and coastal development—coral reefs have been observed to shift from coral- to algae-dominated states [12]. Many mechanistic models have been proposed for hypothesis-testing about mechanisms that drive spatial patterning and long-term resilience [13–18]. In particular, Mumby, Hastings and Edwards (MHE) developed a simple three-species temporal model for Caribbean coral reefs accounting for corals and two types of algae, algal turfs and macroalgae [19]. The MHE model demonstrated that there are coral- and algae-dominated alternative states and established critical thresholds of fish grazing and coral cover delineating the resilience of each state.

A natural question is how coral resilience is affected by the spatial distribution of coral and other species within the reef. To address this question, we build on the MHE model [19] to develop a stochastic and spatial lattice-based model (sMHE) of a coral reef. Stochasticity is essential to model the unpredictable disturbances that affect coral reefs and enables transitions between the alternative stable states identified by the MHE model. Spatial models, both deterministic (based on partial differential equations) and stochastic (e.g. agent-based and cellular automata models), have been used to study pattern formation [4,5,20]. Due to stochasticity, multiple realizations of the sMHE model are required to produce statistical results and develop insight into the reef-level dynamics while retaining the spatial information. To this end, we use topological descriptors suited to averaging over realizations. We first consider neighbourhood descriptors that quantify the clustering of coral throughout the reef and then appeal to TDA.

TDA is a branch of computational mathematics that summarizes the shape of data through topological invariants [21,22]. Persistent homology (PH), a prominent tool in TDA, takes in data and outputs a multiscale topological summary of features, such as connected components, loops and cavities [23]. Depending on the type of data being studied, PH offers a flexible suite of methodologies which may be adapted to address the research question at hand [24]. PH has previously generated insight into many biological applications [25–27]. While competition for space is known to affect reef dynamics [18], much conventional analysis of reef data focuses on the prevalence of different species (measured through percentage cover). We use TDA to analyse simulated and empirical data, due to its ability to capture spatial features and patterning not detected by standard analyses. To perform statistics, classification, comparison and averages of topological features, vectorization methods of PH have been developed [28–30]. Such statistical techniques allow the computation of robust spatial properties of complex, possibly noisy coral reef data.

We compute PH of coral data extracted from photographs of underwater reefs and from snapshots of the sMHE model. However, standard PH is limited to studying static, non-dynamical data. Advances in TDA have enabled the analysis of data that evolve non-monotonically over time [31–34], including the generalization of PH to zigzag persistence [35–37]. As with standard PH, zigzag persistence detects topological features in data such as components, loops and cavities. However, zigzag persistence allows such features to be tracked over multiple time-snapshots, which is not possible with standard PH. We therefore use zigzag persistence to analyse how the spatial composition of reefs changes over time. Vectorization methods created for PH are also applicable to zigzag persistence, and we use these to describe average spatio-temporal properties of coral under different conditions.

Dynamic TDA methods have been previously applied to analysing aggregation models, fish swarms and temporal networks [31,38,39]. However, zigzag persistence of dynamic data for larger systems was computationally out of reach until recently [40]. Here, we show that standard and zigzag PH provide complementary information on species competition in our model and zigzag PH reveals pathways to coral decline.

## 2. Stochastic spatial model and data generation

**Figure 1.**
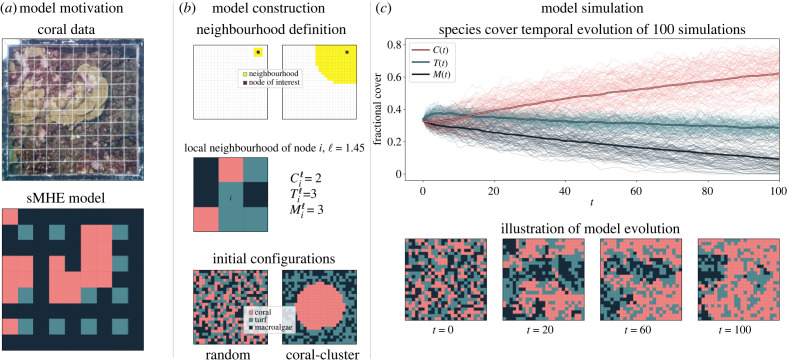
Stochastic spatial sMHE model of a coral reef. (*a*) Model motivation. Quadrat photograph of 1 m^2^ taken on the coral reef in Nuku Hiva (Marquesas Islands, French Polynesia) in 2014, kindly provided by the Service National d’Observation CORAIL from CRIOBE. A 10 × 10 grid mesh is placed over a section of reef, photographed and the species at each grid intersection, i.e. every 10 cm, is noted. Motivated by the image, the sMHE model represents the most prevalent species within each sub-square. (*b*) Model construction. The local neighbourhood of radius ℓ determines a node’s area of influence in the evolution of the model. We show the neighbourhoods of influence corresponding to ℓ = 1.45 and ℓ = 11. The local neighbourhood of node *i* (occupied by turf in the example plotted) has $C_{i}^{\ell }=2$, $T_{i}^{\ell }=3$ and $M_{i}^{\ell }=3$. Given initial covers *C*(0), *T*(0), *M*(0), the sMHE model is initialized either with a *random* configuration, with a uniform distribution representing a spatially mixed reef (any node is initialized as coral, turf or macroalgae, with probability *C*(0), *T*(0), *M*(0), respectively), or with a *coral-cluster* configuration, where the coral is placed in a connected patch at the centre of the domain, with the other two species uniformly distributed in the remaining space. (*c*) Model simulation. Temporal evolution of the model with initial random configuration, ℓ = 1.45, grazing *g* = 0.58, and *C*(0) = *T*(0) = *M*(0) = 0.33, averaged over 100 realizations. Spatial snapshots of the sMHE model are shown at times *t* = 0, *t* = 20, *t* = 60 and *t* = 100 for one realization.

The coral ecosystem is driven through complex interactions between its components and environmental variables, which arise from competition for space and resources [18] (see figure 1*a*, top). In [19], Mumby, Edwards and Hastings proposed the following model (MHE model) to describe such interactions in a simple three-component ODE system:

2.1a $$\dot{C}=rCT-dC-aCM$$

and

2.1b $$\dot{M}=aCM-\frac{gM}{M+T}+\gamma MT.$$

The MHE model describes the temporal evolution of the fraction of a reef covered by either coral (*C*), macroalgae (*M*) or algal turf (*T*), where *T* = 1 − *C* − *M* (assuming the seabed area is fully covered). Previous work has extended the MHE model to account for the effect of ocean acidification [41], natural disasters [42] and more complex fishing dynamics [43,44]. Other models consider multiple competing coral and macroalgae types [45] with complex intra-species dynamics [46]. We use the original MHE model as our starting point due to its simplicity and well-understood dynamics.

In the MHE model (2.1), competition between species is represented by the nonlinear terms, which are agnostic of spatial location. One possible modelling framework which would represent the spatial dependence of coral growth would be a system of partial differential equations (PDEs) describing *C*, *M* and *T* as functions of time and space. However, to account for the complexity of the environment, such inter-species interaction should be stochastic, which would not be reflected in a deterministic PDE. Furthermore, we wish to construct a model which simulates data of the same form as photographed coral reefs overlayed with a grid mesh as in figure 1*a*.

In the stochastic spatial MHE model (sMHE model), we make inter-species competition location-dependent by considering a square-grid discretization of the seabed domain with 25 × 25 nodes, where each node *i* is spatially embedded in two dimensions and occupied by either *C*_*i*_, *T*_*i*_, *M*_*i*_ ∈ {0, 1} (see figure 1*b*, bottom). The number of nodes (625) was chosen to balance simulation time (which increases exponentially with the number of nodes) with the effectiveness of TDA computations (which distinguish more spatial behaviour at finer resolutions). We introduce a local neighbourhood of radius ℓ within which interactions can occur (see figure 1*b*, top). For example, the first term in (2.1*a*) describes the interaction between *C* and *T*, namely the recruitment of coral through the overgrowth of turf at a rate *r*. In the sMHE model, this interaction can only occur if there is a node *i* with turf (for coral to overgrow) within a radius ℓ of a node *j* with coral, that is, *T*_*i*_ = 1, *C*_*j*_ = 1 and |*i* − *j*| ≤ ℓ. The interaction is then represented as the ‘reaction’ *T*_*i*_ + *C*_*j*_ → *C*_*i*_ + *C*_*j*_ with rate *r* (meaning that the transition *T*_*i*_ → *C*_*i*_ occurs with probability *r*Δ*t* within a time interval Δ*t*). The full set of reactions of the sMHE is

2.2a $$T_{i}+C_{j}\overset{r}{\to }C_{i}+C_{j},\;C_{i}\overset{d/\nu_{1}}{⟶}T_{i},\;C_{i}+M_{j}\overset{a}{\to }M_{i}+M_{j}$$

and

2.2b $$M_{i}\overset{g/\nu_{2}}{\to }T_{i},\;T_{i}+M_{j}\overset{\gamma }{\to }M_{i}+M_{j},$$

where *ν*_1_, *ν*_2_ are neighbourhood-dependent functions (see electronic supplementary material, §1, for the full specification of equations (2.2)). Throughout this work, we keep all the model parameters fixed to *r* = 1, *d* = 0.4, *a* = 0.2, *γ* = 0.75 except the neighbourhood radius ℓ and the grazing rate *g* at which fish graze on macroalgae. The fixed parameters are taken from [19]. We initialize the model to initial global densities *C*(0), *T*(0), *M*(0), using either a random initial configuration (uniformly distributed) or a coral-cluster initial configuration (where all coral nodes are placed together in the centre of the domain, and *M* and *T* are uniformly distributed around in the remaining space; see figure 1*b*, bottom). The sMHE model (2.2) is simulated as follows: at fixed time steps Δ*t*, each node *i* is considered in turn and is allowed to react with a probability according to the neighbourhood-dependent rates (see electronic supplementary material, S1, for details on the stochastic simulation algorithm). A simulation of the sMHE model is a sequence of ternary matrices (see figure 1*c*, bottom), which we call snapshots, in which each entry indicates which of the three species occupies that location within the reef. From the matrix, we can extract the global fractional cover of each species at any time, e.g. $C(t)=\sum_{i}C_{i}(t)/625$.

## 3. Descriptors for coral data analysis

**Figure 2.**
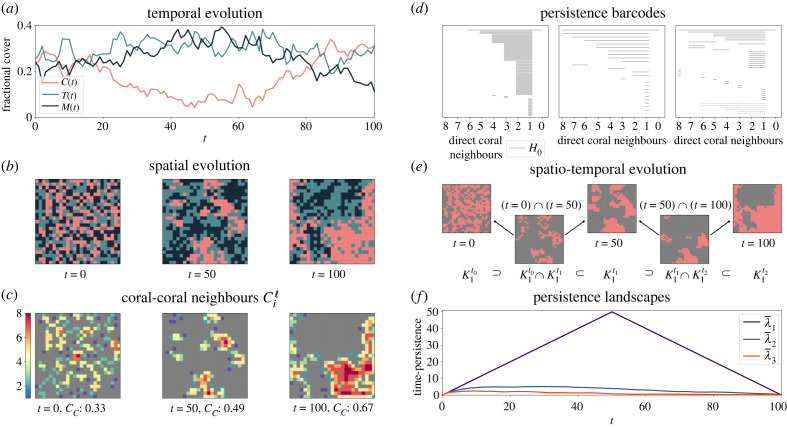
Data analysis of the sMHE model. (*a*) Temporal evolution of fractional covers *C*(*t*), *T*(*t*), *M*(*t*) over 100 timesteps. (*b*) Snapshots of the spatial evolution of the sMHE model at three different times. Colours represent the three species as in figure 1. (*c*) Coral–coral neighbours: heatmaps of $C_{i}^{\ell }$ for each node *i* corresponding to the data in (*b*). (*d*) Persistence barcodes of the three snapshots in (*b*). Solid bars represent clusters; dotted bars represent enclosed loops. The length of the bars represents the sizes of each. The filtration parameter is the number of direct coral neighbours. (*e*) Spatio-temporal evolution: illustration of the zigzag sequence from a single simulation of the sMHE model. We show the three time snapshots in (*b*) (after pre-processing) and their intersections. (*f*) Average persistence landscapes ${\bar{\lambda }}_{1}$, ${\bar{\lambda }}_{2}$, ${\bar{\lambda }}_{3}$, describing the three most time-persistent features in an average simulation of the sMHE model. The first landscape shows there is a single component that lasts throughout all times in all simulations. The second and third landscapes show that, on average, two other components appear early in the simulation and last for a short time.

A simple, effectively non-spatial descriptor is the fractional cover, given by the proportion of nodes of a given type in the reef (figures 1*c* and 2*a*). We explore the spatial dynamics of the sMHE model using a collection of *neighbourhood descriptors*, PH and zigzag persistence.

### 3.1. Neighbourhood descriptors

We introduce nine neighbourhood descriptors to quantify the average neighbourhood composition of nodes in the reef. Let node *i* be of type *C* (*C*_*i*_ = 1). The neighbours of node *i* are those nodes within a radius ℓ of *i* (figure 1*b*, middle). We count the number of neighbours $\sigma_{i}^{\ell }$ of node *i* that are of type *σ* for *σ* ∈ {*C*, *T*, *M*}. We have $\sigma_{i}^{\ell }\in \{0,\ldots \;,n_{i}^{\ell }\}$, where $n_{i}^{\ell }$ is the total number of neighbours of node *i* (e.g. $n_{i}^{1.45}=8$ for an internal node *i*). We then define the neighbourhood descriptors *σ*_*C*_ as the average of $\sigma_{i}/n_{i}^{\ell }$ across all nodes *i* of type *C*. The descriptor *C*_*C*_, for instance, gives the average fraction of coral neighbours that are coral (figure 2*c*). The other six descriptors are defined similarly, considering nodes *i* of type *T* or *M* to give nine descriptors in total (see electronic supplementary material, S1, for details). We may average the fractional coral cover over many realizations (figure 1*c*) to give a non-spatial summary of the model’s behaviour. However, the same coral fractional cover can take many different spatial patterns. For example, the random or coral-cluster initial configurations can be set with the same fractional cover (0.33), yet the local neighbourhood information differs significantly (*C*_*C*_ (random) < 0.2 whereas *C*_*C*_ (coral-cluster) > 0.8) (figure 1*b*, bottom). This local neighbourhood descriptor highlights that coral occupies more than 80% of the neighbours of coral nodes in the coral-cluster configuration but less than 20% for the random configuration, and therefore distinguishes spatially inhomogeneous reefs with the same fractional covers.

### 3.2. Persistence

PH offers an algorithmic way to quantify the connectivity of multiscale data. We represent a snapshot of the sMHE model by a sequence of *cubical complexes*. A cubical complex is a data structure that represents nodes on a grid by vertices and connects adjacent vertices with edges and squares. To encode information about the density of coral nodes, we assign an integer to each vertex *i* with *C*_*i*_ = 1 based on the number of direct coral neighbours. Mathematically, we define a *density filtration function* f : I→N, where I is the set of nodes in the reef and *N* = {0, …, 8} [47,48]. We use cubical complexes and *f* to create a multiscale lens called a *filtration*.

In the first filtration step, we create a cubical complex using only those coral nodes with eight direct coral neighbours (i.e. completely surrounded by coral nodes), adding edges and squares between adjacent coral nodes. At each subsequent step, we include coral nodes with 7, 6, … coral neighbours to the cubical complex. We define a nested sequence of cubical complexes according to the number of direct coral neighbours, $K_{8}\subset K_{7}\subset \cdots \subset K_{2}\subset K_{1}.$

For a given time-snapshot of the sMHE model, we build the filtration and then compute standard PH (see electronic supplementary material, S2, for full details). PH quantifies the topological features, such as clusters *H*_0_ (i.e. connected components) or loops *H*_1_ (i.e. one-dimensional holes) across the filtration. The appearance and disappearance of components and loops across the filtration can be visualized as a multiset of intervals called a *barcode*. Here, barcodes quantify features according to their size since large numbers of direct coral neighbours indicate large clusters (figure 2*c*,*d*). In figure 2*d*, the barcodes capture the temporal evolution of the data, from the random spatial structure described by many short bars at *t* = 0 to the single long bar at time *t* = 100 representing one large coral cluster.

PH considers specific times of the sMHE data independently, making it difficult to decide whether a single component of coral persists or whether different coral clusters appear at each timestep. We want to trace the time evolution of specific spatial features in the sMHE model. Due to the non-monotonicity of coral dynamics (i.e. coral locations can be occupied by other species and then return to coral), standard PH is not suitable, since coral clusters are not nested over time.

### 3.3. Zigzag persistence

To track the evolution of topological features over many time steps, we propose to use zigzag persistence [35], which generalizes the notion of filtration to a *zigzag diagram*. At each time point *t*_*m*_, we choose one of the complexes from the filtration described above to represent a snapshot of the sMHE model. We choose $K_{1}^{t_{m}}$, the cubical complex obtained by including coral nodes with at least one coral neighbour. We then insert the intersection of every pair of successive complexes into this sequence, giving

3.1 $$K_{1}^{t_{0}}⊃(K_{1}^{t_{0}}\cap K_{1}^{t_{1}})\subset K_{1}^{t_{1}}⊃(K_{1}^{t_{1}}\cap K_{1}^{t_{2}})\subset \cdots ⊃K_{1}^{t_{M}}.$$

A diagrammatic representation of (3.1) with three time points is given in figure 2*e*. We use this sequence to compute connected components (i.e. *H*_0_ of (3.1)), which emerge and disappear over many timesteps in a simulation of the sMHE model. We perform a pre-processing step to reduce the noise of the sMHE data before the computation of zigzag persistence (see electronic supplementary material, §3).

Due to the stochastic nature of the sMHE model, the zigzag barcode of a single simulation would not provide an accurate summary of the model’s behaviour. Persistence landscapes offer a way to average the spatial information encoded in barcodes [28]. We create a persistence landscape from a simulation of the sMHE model as follows. For each component of coral that is detected in a simulation of the model, we plot a landscape peak at (*t*_*l*_, *s*_*l*_), where *t*_*l*_ is halfway through the component’s life span, and *s*_*l*_ is half of the length of time for which it exists. For example, if a component is born at time *t* = 50 and dies at time *t* = 100, a landscape peak is placed at (75, 25). A ‘tent’ is then constructed by connecting (*t*_*l*_, *s*_*l*_) linearly down to the birth and death times on the *t*-axis. The *k*th persistence landscape \lambda _k takes, at every time, the *k*th largest value among all the ‘tents’ (see electronic supplementary material, §3, for a full explanation of this process). The landscape ${\bar{\lambda }}_{k}$ is the average of \lambda _k over many simulations, and the maximum of each ${\bar{\lambda }}_{k}$ may be interpreted as the average half-lifetime of the *k*th most persistent cluster. Therefore, zigzag persistence landscapes quantify the typical spatial information across multiple simulations of the sMHE model and rank which features are most significant over time.

The first landscape (${\bar{\lambda }}_{1}$) in figure 2*f* highlights that a single cluster of coral dominates the domain from *t* = 0 through *t* = 100. We can observe that the time-persistence of the second and third landscapes (${\bar{\lambda }}_{2},{\bar{\lambda }}_{3}$) decreases as time increases, suggesting that smaller components either disappear or join with the main one as time increases. Zigzag persistence, therefore, confirms that, on average, a single component is emerging over time, which PH alone cannot conclude.

We use these tools to explore the model dynamics and spatial structure. Specifically, we consider the effect of local neighbourhood radius ℓ, the initial configuration of species (random or coral-cluster configurations), and the rate *g* that fish graze on macroalgae, and quantify how changing these three parameters affects the sMHE model.

## 4. Results

### 4.1. Spatial interactions lead to coral clustering

**Figure 3.**
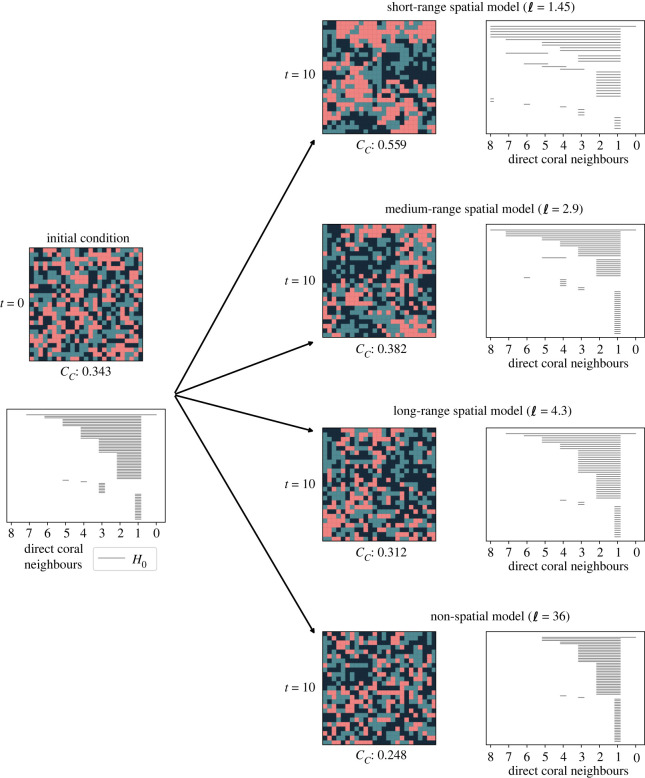
Effect of neighbourhood threshold spatial and non-spatial versions of the sMHE model are compared by running simulations up to time *t* = 10. The grazing rate is kept constant at *g* = 0.53, the random initial configuration is used with equal initial fractional covers *C*(0), *T*(0) and *M*(0). The neighbourhood size is varied: ℓ = 1.45, 2.9, 4.3, 36. As described in figure 1, a neighbourhood size of ℓ = 1.45 gives a spatial model, where only the immediate eight neighbours of each node affect its transition as the model is updated. The values ℓ = 2.9, 4.3 give spatial models where a larger grid of nodes affects the reaction rates. When ℓ = 36, all nodes are considered neighbours of all others, giving a non-spatial model. The snapshots of the sMHE model are printed at time *t* = 0 and at time *t* = 10 for each of these scenarios. The coral–coral neighbourhood descriptor, *C*_*C*_, distinguishes the three cases. The value *C*_*C*_ = 0.559 for the ℓ = 1.45 spatial model indicates that coral clusters more tightly together in this case, whereas the value *C*_*C*_ = 0.248 shows that this does not happen for the non-spatial model. The adjacent persistence barcodes give further details of this difference. For the ℓ = 1.45 spatial model, there are a few large components of coral (indicated by solid bars) and some enclosed loops (indicated by dotted bars). For the non-spatial case, there are many smaller components of coral and no loops. The ℓ = 2.9, 4.3 plots give intermediate results in both *C*_*C*_ and the persistent barcode.

The range of the spatial interactions in the sMHE model is controlled by the neighbourhood radius ℓ (figure 1*b*). For large values of ℓ, the neighbourhood of interaction spans the whole domain, and the sMHE model reduces to a non-spatial stochastic model. However for small values of ℓ the rates of reactions (2.2) are highly dependent on a node’s immediate neighbours. The neighbourhood descriptor *C*_*C*_ and PH describe the spatial patterning observed in simulations for different values of the neighbourhood radius ℓ (see one realization in figure 3). In particular, larger clusters of coral appear when the interaction range ℓ is small, whereas we observe little spatial patterning when ℓ is large. Zigzag persistence determines over multiple timesteps that a stable cluster persists over time in the sMHE model with ℓ = 1.45, whereas no such cluster persists in the non-spatial model (see electronic supplementary material, figure S9).

### 4.2. Zigzag persistence distinguishes initial configurations

**Figure 4.**
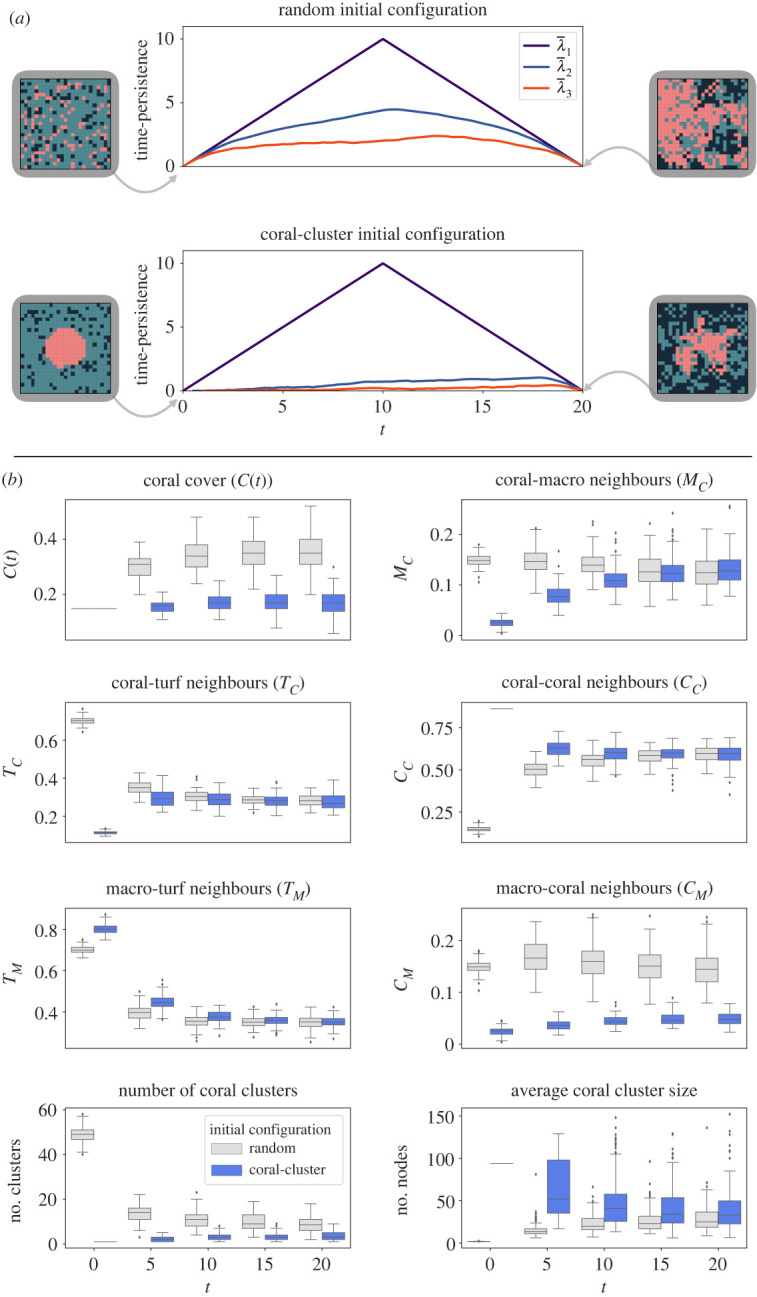
Effect of initial configuration comparison of 100 realizations of the sMHE model initialized with either the random or coral-cluster initial configurations from figure 1*b*, with grazing fixed to *g* = 0.53 and initial fractional covers *C*(0) = *M*(0) = 0.15. (*a*) Average persistence landscapes ${\bar{\lambda }}_{1},{\bar{\lambda }}_{2},{\bar{\lambda }}_{3}$. While the random initial configuration leads to three non-trivial landscapes, the coral-cluster initial configuration gives a significant first landscape ${\bar{\lambda }}_{1}$. These average landscapes indicate that the random initial configuration leads to coral clustering in many different components, which persist as separate patches up to at least time *t* = 20. By contrast, the coral-cluster initial configuration begins with a single connected component of coral. The analysis shows that few other components form throughout the simulation and that coral remains clustered in the initial central patch. Examples of model snapshots at *t* = 0 and *t* = 20 are printed at the side of each landscape plot. (*b*) Coral fractional cover *C*(*t*) and neighbourhood descriptors. The plot of *C*(*t*) shows that the random initial configuration favours coral growth, since the fractional coral cover increases in these simulations. By contrast, coral appears to die out when simulations start with the coral-cluster initial configuration. The *T*_*C*_, *M*_*C*_ and *C*_*C*_ plots show that these descriptors clearly distinguish the two initial configurations at time *t* = 0 but cannot tell them apart after a small number of timesteps. Neighbourhood descriptors give similar values for the two initial configurations from time *t* = 5 onwards.

Intuitively, we may think that a single large coral cluster might offer the best conditions for coral resilience over time. To test this hypothesis, we initialize the sMHE model to two initial conditions with identical fractional covers (*C*(0), *M*(0), *T*(0)) but very different spatial configurations, namely the random and coral-cluster initial configurations (figure 1*b*). Perhaps surprisingly, depending on the grazing conditions, the analysis suggests that coral can be more robust over time when initialized with the random configuration, whereas it stagnates or even becomes extinct when initialized with the coral-cluster configuration. The neighbourhood descriptors, as well as the average cluster size and total number of clusters, distinguish the two initial configurations, showing significant differences between the two cases at *t* = 0. However, these descriptors cannot discern the two cases as time progresses (figure 4*b*). On the other hand, zigzag persistence landscapes ${\bar{\lambda }}_{2},{\bar{\lambda }}_{3}$—averaged over many simulations—significantly differ between the two cases over the whole simulation time (figure 4*a*). We can understand this result by noting that the random initial configuration yields a more significant average number of coral-turf neighbours (*T*_*C*_(0) = 0.765 and 0.113 for random and coral-cluster initial conditions, respectively), increasing the locations where coral growth can occur (via the interaction *T*_*i*_ + *C*_*j*_ → *C*_*i*_ + *C*_*j*_) when compared with the coral-cluster initial configuration. In this way, under certain parameter regimes, the spatial configuration of nodes in the sMHE model may determine the long-term behaviour of the system.

### 4.3. Zigzag persistence describes coral extinction pathway

In many reefs, coral’s spatio-temporal dynamics critically depends on the fish population feeding on the macroalgae [49]. Thriving fish populations (i.e. high grazing rate *g*) keep macroalgae levels low, which leads to better conditions for coral to flourish. By contrast, overfishing and natural disasters shrink the fish population and hence *g*, which may result in macroalgae overgrowth and coral decay.

The MHE model (2.1) captured these coral-reef interactions and established that the grazing rate *g* is a bifurcation parameter, where low grazing leads to coral extinction, and intermediate grazing drives the system to display two alternative states [19].

The sMHE model (2.2) reproduces this behaviour: for *g* < 0.52 the system evolves to a macroalgae-dominated reef; for *g* > 0.55 the system evolves to a coral-dominated reef. In the region of multistability (*g* ∈ [0.52, 0.55]), we observe that simulations can evolve to either the coral- or macroalgae-dominated state. However, in contrast to original MHE model, where the long-term behaviours of simulations in the metastable region are determined by their initial condition, the stochastic nature of the sMHE model means that identical initial conditions may lead to different outcomes. At *g* = 0.53, we observed that around half of all simulations (initiated with equal numbers of coral, turf and macroalgae nodes in the random configuration) evolved to the macroalgae-dominated state, with the other half converging to a coral-dominated state.

Metastability implies that the system takes a long time to converge to such a state (up to 1000 timesteps in some realizations). We explore whether summaries of the early species’ behaviour (we choose *t* ∈ [0, 100]) predict the system outcome. Non-spatial descriptors (e.g. the fractional covers *C*(*t*), *M*(*t*); see electronic supplementary material, figure S11) can give an early indication of the system outcome in the metastable region.

But what makes coral die out in some runs of the sMHE in the metastable region and resist in others?

**Figure 5.**
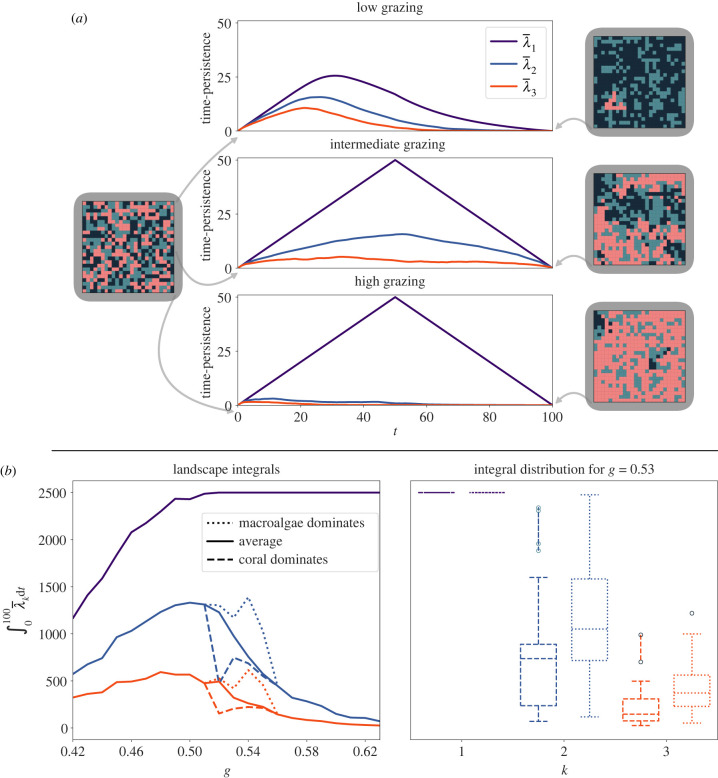
Effect of grazing parameter. (*a*) Average persistence landscapes using 100 realizations of the sMHE model up to *t* = 100 for three grazing rates: *g* = 0.42 (low grazing), *g* = 0.53 (intermediate grazing) and *g* = 0.62 (high grazing). All realizations are initialized with the same initial configuration (pictured near *t* = 0). Representative outputs of the sMHE model at *t* = 100 for each grazing rate are also shown. The integrals of ${\bar{\lambda }}_{k},k=1,2,3$ are computed for each grazing rate. The peak of the first landscape increases as the grazing rate increases. The increasing size of the first landscape indicates that the most resilient component of coral lasts for a longer time for higher, coral-favouring grazing rates. The second landscape is small for low *g* since all components of coral are short-lived in these conditions, as macroalgae quickly overgrow them. On the other hand, the second landscape is small for large *g* as well, because smaller components of coral are quickly amalgamated into the main component in these conditions, as coral begins to dominate the entire domain. These phenomena are balanced for *g* in the middle of this range, where either coral or macroalgae may be successful. Zigzag persistence, therefore, not only describes the change in spatial behaviour as *g* is increased, it indicates that the ‘tipping point’—where coral and macroalgae are evenly matched—corresponds to the peak of the second landscape ${\bar{\lambda }}_{2}$, which occurs near *g* = 0.51. (*b*) The average integrals, between *t* = 0 and *t* = 100, of the *k*th landscapes for different grazing rates. The maximum integral of ${\bar{\lambda }}_{2}$ coincides with the metastable region. Values of these integrals are plotted separately for simulations where coral ended up dominating the reef from those where macroalgae dominated. The integrals of ${\bar{\lambda }}_{2}$ and ${\bar{\lambda }}_{3}$ are greater in cases where coral dies out, indicating that it does so through many short-lived components rather than the principal component.

To address this question, we turn to zigzag persistence. Zigzag persistence can predict the system’s outcome (figure 5*a*) but also characterizes how the coral’s spatial clustering affects coral outcome (figure 5*b*). In particular, we find that ${\bar{\lambda }}_{2}$ and ${\bar{\lambda }}_{3}$ (corresponding to the second and third most dominant topological features) are higher in simulations where coral eventually dies out. The difference in landscape size for different outcomes indicates that under these reef conditions (in particular, low coral-turf neighbours, *T*_*C*_(0) = 0.361), coral extinction occurs through multiple small clusters (as opposed to one cluster that shrinks to nothing), while coral domination establishes itself as one persistent cluster.

### 4.4. Topology distinguishes empirical data with same coral cover

**Figure 6.**
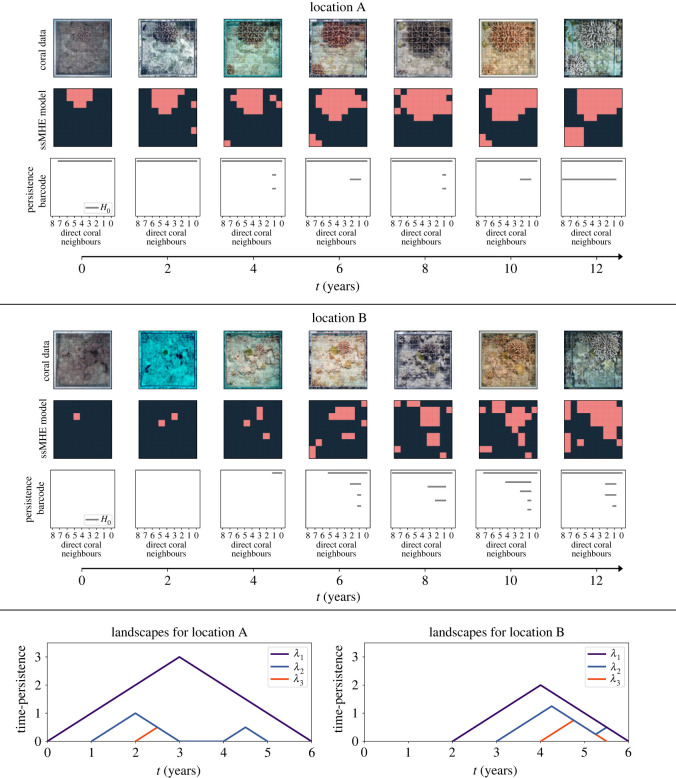
Analysis of empirical data. Comparison of two multi-year coral datasets at different locations within the Rarotonga reef (Cook Islands) from 2009 to 2022. Data were kindly provided by the Service National d’Observation CORAIL from CRIOBE. Locations A and B. For each location, we show a 1 m^2^ section of reef with a 10 × 10 grid placed on top, photographed on seven different occasions over a 12-year period. We estimate (by eye) the grid intersections where coral is present and use this to represent each photograph by a snapshot of the sMHE model. Pink squares denote locations where we see coral and all other squares are coloured blue. We then compute persistence barcodes of the model snapshots corresponding to each photograph at each location. In location A, all barcodes contain one persistent bar, with some containing additional short bars. This indicates that one large component of coral is present within each photograph of location A, as well as some small components at certain timesteps. On the other hand, the barcodes corresponding to the photographs of location B show many short bars and one long bar—with the longest bar increasing in length as time goes on. This suggests that there is principal component of coral in location B which is getting larger. Landscapes for both locations. The zigzag landscapes for each location provide additional information to the persistence barcodes and distinguish the spatio-temporal evolution in each location. In location A, the first landscape indicates that a single component of coral exists throughout all photographs. The second and third landscapes show short-lived components of coral which either disappear or join to the principal component. The peak of the first landscape is smaller and occurs later for location B, indicating that the principal component emerges towards the end of the study period. While the percentage cover of coral (≈40%) and the *C*_*C*_ neighbourhood descriptor (≈0.75) are similar at the two locations at the final timestep, the zigzag persistence landscapes clearly distinguish the spatio-temporal evolution of coral at the two locations.

We next use the longitudinal spatial data from the Moorea IDEA project [50] to exemplify the use of the proposed topological descriptors. Our methods would be ideally suited for a grid with finer spatial resolution, but data of coral reefs via drones [51] which offer higher spatial resolution currently lack multi-year collection. Here, we apply our topological methods to two multi-year datasets from the Rarotonga reef at two different locations (see figure 6, locations A and B). While the percentage cover of coral is similar at the start (*t* = 0) and at the end (*t* = 12) of the 12-year period for both locations, persistence barcodes and zigzag landscapes distinguish the spatio-temporal evolution (see figure 6, landscapes).

The coral configurations in the two locations (A and B) resemble the coral-cluster and random configurations of the sMHE model, respectively. Similar to the model analysis in figure 4, the coral–coral neighbourhood descriptor (*C*_*C*_) is the same for both locations at the end of the time-course. However, the persistence barcodes and zigzag landscapes differ. Barcodes from the empirical coral-cluster (figure 6, location A) have one persistent bar for each time iterate, whereas the dataset analogous to the random configuration (figure 6, location B) has many short bars in the early iterates and one long bar at the final timestep. The spatial–temporal evolution at different locations can be distinguished by the zigzag landscapes (figure 6, landscapes). In location A, the first zigzag landscape is larger than for location B, and the peaks of landscapes for location B occur later than for location A. The topological analysis therefore reveals that, in location A, one large cluster persists across twelve years, whereas in location B, many small clusters join together over time. Our model predicts that configurations similar to location A are more likely to lead to coral extinction than those similar to location B. To justify this prediction, we would require empirical reef data with better resolution (spatial and temporal). In future, we aim to compare data with coral evolution models such as the sMHE model.

## 5. Discussion

Motivated by ecology and evolution, as well as the increasing availability of spatial data of such processes, we introduced a stochastic spatio-temporal lattice-based model (sMHE model (2.2)) of coral reefs. We collected data through computational experiments and proposed topological descriptors to quantify coral behaviour and predict mechanisms. Specifically, we explored these descriptors on multiple realizations of coral reef dynamics under changes to the initial configurations and the values of key model parameters.

The evolution of the sMHE model depends on two factors. First, species evolve based on the rate values in equations (2.2) (which model internal and external factors affecting the reef as in the original MHE model [19]) and, second, on the spatial arrangement of nodes in the reef. A combination of both factors determines which reactions are possible (depending on the make-up of the neighbourhood) and more likely to occur. The fish grazing rate *g* is a critical parameter in both the MHE and sMHE models, with macroalgae dominating for low enough *g* and coral dominating for high enough *g* (regardless of the spatial arrangement of species in the reef). By contrast, in the intermediate metastable region, the coral- and macroalgae-favouring reactions balance out so that the spatial arrangement becomes a deciding factor. Zigzag persistence can discern these multiple pathways, intricately dependent on species’ competition. For example, we found that the random initial configuration yields higher coral growth than the coral-cluster initial configuration (figure 4) under turf abundance. Conversely, when macroalgae growth overwhelms coral, zigzag persistence suggests that coral goes extinct by becoming dissected into many components (figure 5*b*). This indicates that, when macroalgae prevalence is high, coral survives better when clustered together, thus limiting the macroalgae-overtaking-coral interaction in (2.2). Together, these insights show the potential use of our model in helping assess the vulnerability of reefs and better design artificial reefs. By tweaking our model to the conditions of a reef of interest, one can theorize coral’s ideal spacing for survival.

The region of metastability of the sMHE model (in which different outcomes are possible) results from including spatial and stochastic dynamics in our model. This variation in outcomes motivates questions such as: are there disturbances or interventions that could ‘shock’ reefs into one state or another? Could this region make the fate of reefs more ‘reversible’ than one would assume in the analysis of deterministic models? Could this behaviour explain some of the ‘noise’ observed in real data, with high coral mortality in some locations and coral survival in others with similar conditions?

The advantage of topological spatial descriptors is their versatility in analysing spatial and temporal structure in complex data. With the flexibility of PH to propose and adapt different filtrations, the standard PH pipeline can be tailored to study a wide range of other spatially patterned systems. Here we showcased the power of zigzag persistence, a topological measure that has recently benefited from improved computation [40]. We combined zigzag persistence with statistical landscapes [28] to enhance the identification of the geometry of the initial configuration of species as well as the geometric mechanisms of species competition and, ultimately, coral extinction in the metastable parameter region.

Since zigzag persistence quantifies spatial patterns in images, it can be used as a pre-processing step prior to classification. For example, if using data sources such as drones or satellite images, one may vectorize persistence using persistence landscapes, which can then be fed into machine learning algorithms. Based on the positive findings from analysing short-time duration data generated by the sMHE model, a promising future direction is to apply topological descriptors to such high-resolution, short-time duration drone data. Given such highly resolved data, we expect the classification, annotation and localization of species in images with machine learning and topological statistics will become automatic.

We have found that PH and zigzag persistence provide valuable insights into the spatial dynamics of the sMHE model. Topological quantification has been used to describe complex biological models, with different filtrations and topological descriptors used depending on the specific model in question [25,52,53]. Such topological analyses may, in future, enable the comparison and validation of complex mechanistic models by adapting topological approximate Bayesian computation [54].

## Data Availability

All simulations of the sMHE model were performed in Python: https://github.com/rneuhausler/coralModel-TDA-study. The neighbourhood descriptors are calculated while running the model, as the neighbourhood information is used in the model’s evolution. Computation of PH and zigzag persistence was implemented in Python using the BATS package: https://github.com/CompTop/BATS.py. Code for all figures is available at https://github.com/rmcdomaths/zigzagcoralmodel. All data used in this project may be simulated by running scripts available from the GitHub repository: https://github.com/rneuhausler/coralModel-TDA-study. Alternatively, the data may be downloaded directly from https://doi.org/10.6084/m9.figshare.23717409.v1. Electronic supplementary material is available [55].
